# Mysteries, Epistemological Modesty, and Artificial Intelligence in Surgery

**DOI:** 10.3389/frai.2019.00032

**Published:** 2020-01-21

**Authors:** Tyler J. Loftus, Gilbert R. Upchurch, Daniel Delitto, Parisa Rashidi, Azra Bihorac

**Affiliations:** ^1^Department of Surgery, University of Florida Health, Gainesville, FL, United States; ^2^Departments of Biomedical Engineering, Computer and Information Science and Engineering, and Electrical and Computer Engineering, University of Florida, Gainesville, FL, United States; ^3^Department of Medicine, University of Florida Health, Gainesville, FL, United States

**Keywords:** surgery, machine learning, artificial intelligence, informed consent, risk prediction, phenotyping, prognostics, decision-making

Life is filled with puzzles and mysteries, and we often fail to recognize the difference. As described by Gregory Treverton and Malcolm Gladwell, puzzles are solved by gathering and assimilating all relevant data in a logical, linear fashion, as in deciding which antibiotic to prescribe for an infection. In contrast, mysteries remain unsolved until all relevant data are analyzed and interpreted in a way that appreciates their depth and complexity, as in determining how to best modulate the host immune response to infection. When investigating mysteries, we often fail to appreciate their depth and complexity. Instead, we gather and assimilate more data, treating the mystery like a puzzle. This strategy is often unsuccessful. Traditional approaches to predictive analytics and phenotyping in surgery use this strategy.

## Weaknesses Inherent to Traditional Predictive Analytics and Phenotyping

Postoperatively, most patients recover along a clinical trajectory that can be predicted by their physiologic reserve, the severity of the underlying disease process, and the physiologic insult associated with the planned operation. These predictions augment the decision to offer an operation and inform discussions with patients and their caregivers regarding treatment options and prognosis. This process often relies on biased, error-prone individual judgement, especially when decisions are made under time constraints and uncertainty, leading to preventable harm. Decision-support tools are intended to augment this process. Unfortunately, traditional decision-support tools regard postoperative trajectories as puzzles which may be solved by gathering and assimilating relevant data in a logical, linear fashion with parametric regression modeling. Some regression models predict dichotomous outcomes with accuracy similar to a coin toss. For example, in applying six different regression-based prediction models to 1,380 patients undergoing colorectal surgery, Bagnall et al. (2018) found that all six models performed poorly with area under the receiver operating characteristic curve (AUROC) 0.46–0.61 (Bagnall et al., 2018). In these cases, poor model accuracy is often attributed to stochastic, or random, risk.

## Stochastic Risk and Epistemological Modesty

For surgeons who are sometimes wrong but never in doubt, stochastic risk is an uncharacteristic foray into epistemological modesty, or recognition that our knowledge and understanding are limited. However, if what we call stochastic risk is instead risk that we have failed to predict because we are treating mysteries like puzzles and using the wrong prediction tools, then we are exercising ignorance and complacency, not epistemological modesty. Parametric regression models make predictions with logical, linear rules expressed as algorithms; machine and deep learning artificial intelligence models accurately represent the complex, non-linear associations among inputs and outputs by learning from examples. Because pathophysiology does not consistently conform to additive, linear rules, one might expect that artificial intelligence models would be advantageous.

## Advantages for Artificial Intelligence in Predictive Analytics and Phenotyping

For some tasks, like predicting mortality among heart failure patients, logistic regression can perform as well or better than certain machine learning methods like regression tree analysis (Austin et al., 2010). For complex tasks like predicting several postoperative complications, artificial intelligence models outperform regression-based techniques and clinician judgement (Bertsimas et al., 2018; Bihorac et al., 2018; Brennan et al., 2019). Bertsimas et al. (2018) developed an Optimal Classification Trees machine learning model to predict mortality and 18 complications following emergency surgery, demonstrating superior accuracy compared with the ACS NSQIP calculator (AUROC 0.92 vs. 0.90). The online and phone application asks users 4–11 questions that are generated in response to prior answers. Manual data entry requires more time and input from providers than an automated model, but obviates requirements for data security and encryption of protected health information from electronic health records (EHR). Bihorac et al. (2018) developed and validated the *MySurgeryRisk* platform with automated EHR data linked to US Census data regarding neighborhood characteristics, using 285 variables to predict eight postoperative complications with AUROC 0.82–0.94 (Bihorac et al., 2018). EHR data feeds the algorithm automatically, obviating manual data search and entry, and overcoming a major obstacle to clinical adoption. In a prospective usability study, algorithm accuracy was significantly greater than physician accuracy in predicting postoperative complications (Brennan et al., 2019). These observations have profound implications for the complex, high-stakes decisions surgeons make when offering an operation and addressing modifiable risk factors, tasks that are currently supported by the National Surgical Quality Improvement Program (NSQIP) Surgical Risk Calculator. If machine learning methods consistently outperform the NSQIP calculator and individual surgeon judgement, then surgeons will face a professional and moral imperative to integrate machine learning in the shared decision-making process of informed consent.

Risk assessments and predictive analytics depend on phenotyping to accurately identify and classify patients, diseases, and complications. Phenotyping is also critically important for identifying candidates for emerging treatments and clinical trial enrollment and standardizing definitions for clinical and research applications. Similar to traditional predictive analytics, traditional phenotyping uses rules expressed as algorithms, gathering data into additive and parametric models, treating classification tasks as puzzles. Results from this approach are highly variable, particularly for complex conditions like frailty. Flaatten and Clegg (2018) demonstrated that frailty phenotyping is highly variable among critically ill patients from different institutions—even when applying a single, validated instrument to each individual cohort—with the incidence of frailty ranging from 13 to 53%, without discernable trends relating frailty to chronological age. Surgeons and their patients need accurate frailty phenotyping to inform the decision to operate, identify patients who may benefit from prehabilitation prior to major elective surgery, and predict the likelihood of postoperative complications and recovery (Barberan-Garcia et al., 2018). Emerging evidence suggests that even relatively common and highly morbid conditions with established international consensus definitions like the acute respiratory distress syndrome (ARDS) have subtypes that impact management strategies and outcomes, but are often unrecognized. Sinha and Calfee (2019) found that combining clinical and biological data can identify hyper- and hypo-inflammatory ARDS phenotypes that have different responses to mechanical ventilation strategies, intravenous fluid management, and medications. Notably, suboptimal identification and classification of ARDS may portend failure to rescue postoperative patients (Ghaferi et al., 2009).

**Figure 1:**
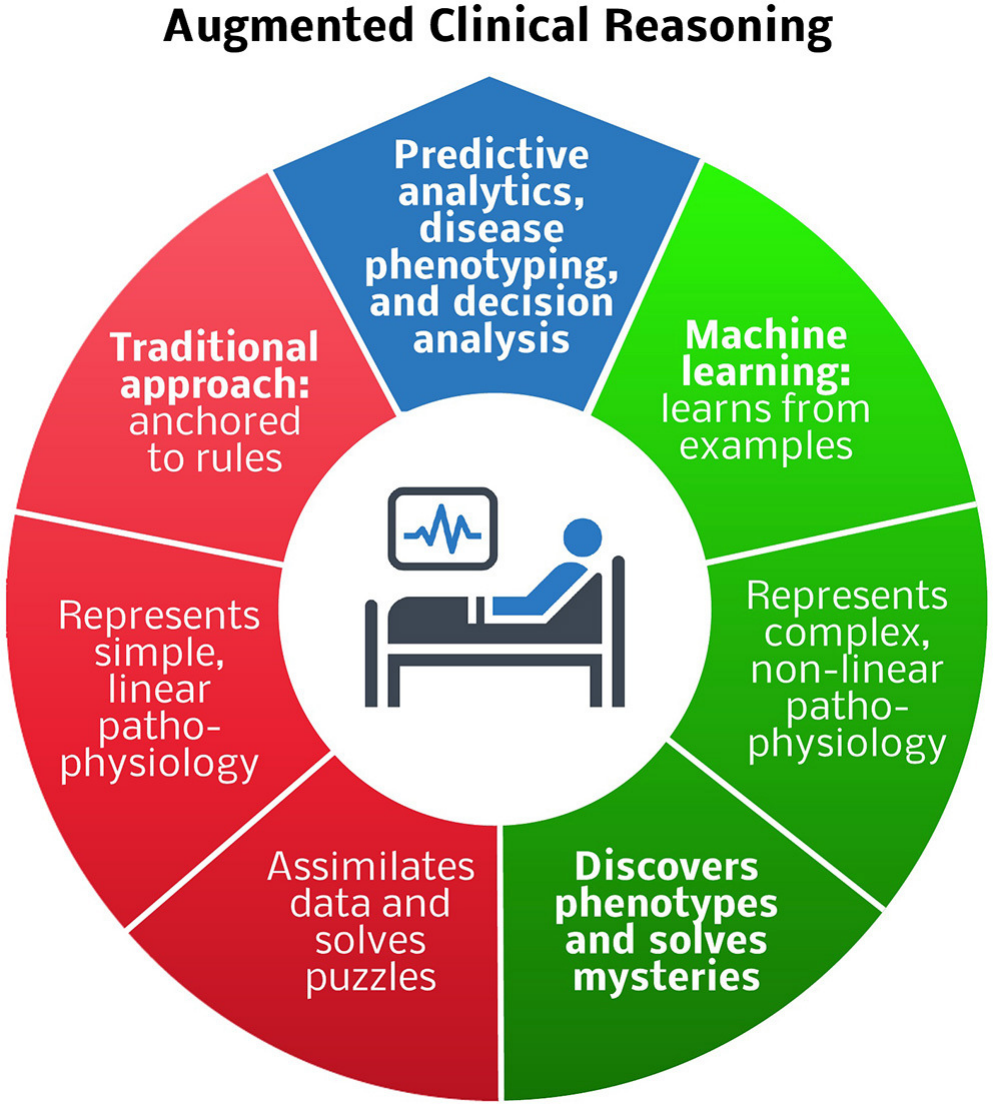
Comparison of traditional and machine learning approaches to predictive analytics, disease phenotyping, and decision analysis for augmented clinical reasoning. Blue region: predictive analytics and disease phenotyping are important tasks in patient-centered decision-making. Red region: traditional rule-based approaches are adequate for representing simple, linear pathophysiology, and solving puzzles, e.g., deciding which antibiotic to prescribe for an infection. Green region: machine learning techniques that learn from examples are preferable for representing complex, non-linear pathophysiology, and solving mysteries, e.g., determining how to best modulate the host immune response to infection.

As an alternative to traditional phenotyping methods, deep learning models can autonomously and accurately phenotype according to established definitions. In addition to performing predictive analytics, facilitating clinical trial enrollment, and standardizing definitions for clinical and research applications, deep learning can solve phenotyping mysteries. Unsupervised models learn relationships and concepts from data and identify patterns and clusters, promoting the discovery of new clinically relevant phenotypes. Artificial intelligence has the potential to revolutionize oncologic phenotyping and prognostication. Among patients with pancreatic cancer, circulating tumor cell histopathology independently predicts the timing of disease recurrence as well as overall survival when adjusting for margin status and tumor grade (Poruk et al., 2016). The clinical utility of this observation is subject to the time-consuming and resource-intense nature of performing and interpreting immunohistochemistry. Alternatively, computer vision programs are adept at performing similar tasks, like recognizing skin cancer with greater accuracy than board-certified Dermatologists, producing results in moments (Esteva et al., 2017). This approach could be used to inform decisions regarding systemic therapies for cancer patients. Kather et al. (2019) demonstrated that deep learning can accurately detect tumor (AUC < 0.99) and predict microsatellite instability on hematoxylin and eosin-stained slides (AUC 0.77–0.84), identifying patients who are likely to benefit from immunotherapy. By learning from examples and representing complex, non-linear pathophysiology, machine learning has the potential to augment clinical reasoning in surgery by performing predictive analytics, and disease phenotyping, and decision analysis tasks that are beyond the reach of traditional methods (Figure 1).

## Challenges and Solutions for Artificial Intelligence Applications in Surgery

Despite these advantages, machine learning models have several limitations that must be addressed prior to widespread clinical adoption. Clinicians may be unfamiliar with methods for interpreting machine learning outputs. Conventional methods like Random Forest variants are relatively transparent and easy to interpret, and emerging techniques improve the interpretability of deep learning models, but it remains difficult to ascertain the relative importance of individual model inputs in determining outputs. To improve output interpretability, model self-attention mechanisms can reveal periods during which inputs make significant contributions to outputs, and models can be trained on labeled patient data and then a linear gradient boosting tree so that the model will assign relative importance to patient data input features (Che et al., 2016; Shickel et al., 2019). However, many clinicians also have difficulty interpreting regression outputs like odds ratios, relative risk values, and even simple *p*-values, suggesting that improving statistical fluency is a global objective that is not unique to artificial intelligence modeling (Anderson et al., 2013; Krouss et al., 2016).

Machine and deep learning models perform well, but like regression models, they are fallible. When they fail, they could impact a large number of patients in a short period of time. Therefore, careful monitoring of model outputs and interpretation by astute clinicians is critically important. Artificial intelligence models are capable of providing a proxy measure of how confident they are that their output is accurate, which can alert clinicians to situations in which outputs should not be trusted. This confidence level can be approximated using an activation function on the final layer of a machine learning model with a softmax function that maps network activations to (0,1), with lower values suggesting lower confidence that predicted probabilities match true probabilities, and higher values suggesting higher confidence. Notably, a model may be uncertain of its predictions even when the softmax output is high (Gal, 2016). Alternatively, the predicted probabilities of machine learning models may be calibrated with reliability curves, producing confidence scores rather than distributions of possible outputs (Guo et al., 2017).

In addition, ethical challenges may arise when models fail and liability is distributed among computer programs, their developers, and the clinicians using the programs. Surgeons, data scientists, informatics experts, and ethicists must work together to address these challenges by improving model transparency, optimizing model accuracy, and establishing a framework to assign liability for errors. Initial prospective implementation of artifical intelligence models in surgery should occur on a small scale under close monitoring, consistent with guidelines regarding the Software as Medical Device (SaMD) category created by the US Food and Drug Administration and the International Medical Device Regulators Forum. As technologies continue to improve over time and involved parties commit to thoughtful and sober implementation of these technologies, the safety and efficacy of artificial intelligence healthcare applications will continue on an upward trajectory.

Finally, it seems unlikely that capitalizing on these advantages will be as simple as switching from basic regression-based to machine learning models. Clinical integration of machine learning will require not only extensive medical domain knowledge founded in basic and translational research, but also informatics expertise, multidisciplinary collaboration, and skillful application of implementation science.

## Conclusions

True epistemological modesty recognizes that continued reliance on individual judgement and traditional predictive analytics and phenotyping may lead to preventable harm. It is irresponsible to attribute these failings to mysterious pathophysiology and stochastic processes without deploying new technologies that capture the depth and complexity of underlying pathophysiology and improve phenotyping and predictive accuracy. Thoughtful clinical integration of artificial intelligence has the potential to transform surgical care by augmenting the decision to operate, informing discussions with patients and their caregivers regarding treatment options and prognosis, predicting treatment response to emerging and experimental treatments, and addressing other unsolved mysteries in surgery.

## Author Contributions

TL and DD contributed to conceptual design, performed the literature review, and drafted the manuscript. GU, PR, and AB contributed to conceptual design, interpreted the literature, and made critical revisions.

### Conflict of Interest

The authors declare that the research was conducted in the absence of any commercial or financial relationships that could be construed as a potential conflict of interest.
